# Multivariate analysis and model building for classifying patients in the peroxisomal disorders X-linked adrenoleukodystrophy and Zellweger syndrome in Chinese pediatric patients

**DOI:** 10.1186/s13023-023-02673-x

**Published:** 2023-05-02

**Authors:** Zhixing Zhu, Georgi Z. Genchev, Yanmin Wang, Wei Ji, Xiaofen Zhang, Hui Lu, Sira Sriswasdi, Guoli Tian

**Affiliations:** 1 Shanghai Engineering Research Center for Big Data in Pediatric Precision Medicine; Center for Biomedical Informatics, Shanghai Children’s Hospital; School of Medicine, Shanghai Jiao Tong University, Shanghai, China; 2 Center of Excellence in Computational Molecular Biology, Faculty of Medicine, Chulalongkorn University, Bangkok, Thailand; 3 Newborn Screening Center, Shanghai Children’s Hospital; School of Medicine, Shanghai Jiao Tong University, Shanghai, China; 4 SJTU-Yale Joint Center for Biostatistics, Department of Bioinformatics and Biostatistics, Shanghai Jiao Tong University, Shanghai, China

**Keywords:** X-linked adrenoleukodystrophy, X-ALD, Zellweger syndrome, Very long chain fatty acids, C26: carnitine, Hexacosanoylcarnitine, Newborn screening, Metabolomic signature, PLS-DA, Sparse PLS-DA, PCA, t-SNE

## Abstract

**Background:**

The peroxisome is a ubiquitous single membrane-enclosed organelle with an important metabolic role. Peroxisomal disorders represent a class of medical conditions caused by deficiencies in peroxisome function and are segmented into enzyme-and-transporter defects (defects in single peroxisomal proteins) and peroxisome biogenesis disorders (defects in the peroxin proteins, critical for normal peroxisome assembly and biogenesis). In this study, we employed multivariate supervised and non-supervised statistical methods and utilized mass spectrometry data of neurological patients, peroxisomal disorder patients (X-linked adrenoleukodystrophy and Zellweger syndrome), and healthy controls to analyze the role of common metabolites in peroxisomal disorders, to develop and refine a classification models of X-linked adrenoleukodystrophy and Zellweger syndrome, and to explore analytes with utility in rapid screening and diagnostics.

**Results:**

T-SNE, PCA, and (sparse) PLS-DA, operated on mass spectrometry data of patients and healthy controls were utilized in this study. The performance of exploratory PLS-DA models was assessed to determine a suitable number of latent components and variables to retain for sparse PLS-DA models. Reduced-features (sparse) PLS-DA models achieved excellent classification performance of X-linked adrenoleukodystrophy and Zellweger syndrome patients.

**Conclusions:**

Our study demonstrated metabolic differences between healthy controls, neurological patients, and peroxisomal disorder (X-linked adrenoleukodystrophy and Zellweger syndrome) patients, refined classification models and showed the potential utility of hexacosanoylcarnitine (C26:0-carnitine) as a screening analyte for Chinese patients in the context of a multivariate discriminant model predictive of peroxisomal disorders.

**Supplementary Information:**

The online version contains supplementary material available at 10.1186/s13023-023-02673-x.

## Introduction

The peroxisome is a ubiquitous single membrane-enclosed organelle which plays important metabolic functions, such as the β-oxidation of very long chain fatty acids, α-oxidation of branched chain fatty acids, synthesis of bile acids and ether-linked phospholipids, and removal of reactive oxygen species [1]. Peroxisomal disorders represent a class of medical conditions caused by defects in peroxisome functions and can be broadly segmented into enzymes and transporter defects (defects in single peroxisomal proteins important for peroxisome function) and peroxisome biogenesis disorders (defects in the peroxins - proteins which are critical for normal peroxisome assembly and biogenesis) [2, 3].

Zellweger syndrome (ZS) (OMIM: 214,100, ICD: Q87.82) is a rare congenital peroxisome biogenesis disorder, the most severe of the four disorders of the Zellweger spectrum, with an estimated prevalence of 1:50,000 [4]. It is characterized by a lack of functioning peroxisomes in the cells of the afflicted individual and is associated with deficient neuronal migration and neuronal positioning, and impairment in the individual’s brain development. The patient may present with high forehead, hypoplastic supraorbital ridges, epicanthal folds, midface hypoplasia, and other craniofacial abnormalities. ZS etiology is due to mutations of the PEX gene family, and the disease has rapid progression and high mortality rate. At present, there are limited options for effective treatment, and treatment options are focused on improvement of quality of life and support.

X-linked adrenoleukodystrophy, X-ALD (OMIM: 300,100, ICD: E71.33) is a rare disease with estimated prevalence of 1 in 15,000 individuals caused by mutations in ABCD1 - an X chromosome gene (Xq28) which codes for the ALD protein. Functional deficiencies in this peroxisomal membrane transporter protein causes buildup of saturated very long-chain fatty acids (VLCFA) in plasma and tissues [5]. The clinical disease course of X-ALD in male patients commences with being asymptomatic at birth. Addison’s disease is often the first display of the disorder that can present years before the appearance of neurological symptoms. The cerebral type of X-ALD can manifest in childhood (childhood cerebral ALD, CCALD), adolescence (adolescent cerebral ALD, AdolCALD) or adulthood (adult cerebral ALD, ACALD) and progress rapidly with deteriorating condition of the patient. Nearly all male patients and the majority of female patients who reach adulthood eventually develop adrenomyeloneuropathy (AMN) [6]. Depending on the specific X-ALD phenotype, patients may present with adrenocortical insufficiency, rapid decline in cognitive abilities, hyperactivity, spastic paraparesis and seizures, to name a few.

Despite their originally shown promise, Lorenzo’s oil and lovastatin have failed to deliver strong evidence as an effective therapy for AMN] [7–9]. Allogeneic bone marrow transplantation or hematopoietic stem cell transplantation are the most effective treatment in cerebral ALD, which if administered at an early time point before neurological symptom appear, can arrest the progression of X-ALD and stop demyelination. The prognosis is poor for advanced stage cerebral X-ALD [10, 11] and alternatively to allogeneic stem cell transplants, therapy involving infusion of autologous CD34 + cells transduced with the elivaldogene tavalentivec (Lenti-D) lentiviral vector [12–14] have been performed on X-ALD patients with effective outcome. Genetic therapy with in- vivo genetic transfer mediated by adeno-associated virus 9 (AAV9) has been developed and shown therapeutic promise [15]. Methods which focus on correction of the endogenous ABCD1 gene in vivo such as homology-independent targeted integration (HITI) [16] or treatment with a direct intracerebral injection of lentiviral ABCD1 [17] may become a treatment paradigm for X-ALD patients in the future. At present however, the temporal window of opportunity for treatment administration is limited, which underscores the necessity of early diagnosis, routine newborn screening, and periodic MRI examinations in order to achieve a favorable prognosis in X-ALD [18].

When there is suspicion of peroxisomal disorder in the patient, the diagnosis is confirmed by biochemical tests, metabolomic profiling, and in recent years by targeted gene sequencing. Such clinical processes are time consuming and labor intensive and underscore the necessity of ongoing further development and improvement of suitable biomarkers [19, 20] that can be utilized in newborn screening, rapid diagnostics, and to avoid missing the therapeutic window for effective treatment. Recent computation effort [21] applied machine learning to establish diagnostic thresholds of clinical biomarkers for differential diagnosis in peroxisomal disorders based on the patient’s VLCFA pattern profile. Overall, while significant progress has been made, unanswered questions remain and integrating available metabolomics datasets with multivariate analysis methods has the potential to enhance the study of peroxisomal disorders for improved screening and diagnostics modalities.

Previously, we explored the utility of tandem mass spectrometry in screening X-ALD and ZS and analyzed and established the reference intervals of a panel of 8 very long-chain lysophosphatidylcholines and acylcarnitines and their ratios for screening of X-ALD patients [22]. Furthermore, we evaluated the concentration level differences of these metabolic features in healthy controls, X-ALD, and ZS patients, and a preliminary metabolic feature subset as a targeted metabolite panel [23] was proposed. The motivation of the present work was to investigate and assess the performance of multivariate modeling and analysis in classifying patient samples in peroxisomal disorder (ZS and X-ALD), non-peroxisomal disorder neurological patients and healthy controls, and to determine models with most improved screening utility. We aimed to analyze the classification potential of very long chain acylcarnitines (VLCAC) and lysophosphatidylcholines (LPC) [24] metabolite data. Our additional goal was refining and augmenting the evidence supporting the VLCAC- and LPC-based features with the most classification potential, and to improve and simplify the accurate screening and diagnosis of peroxisomal disorders (ZS and X-ALD) in pediatric patients and serve as a reference point in the implementation of future clinical diagnosis methods.

## Materials and methods

### Summary

In this study we employed multivariate supervised and non-supervised statistical methods (t-SNE, PCA, PLS-DA, and sparse PLS-DA) operated on mass spectrometry data of neurological patients, peroxisomal disorder (X-linked adrenoleukodystrophy and Zellweger syndrome) patients, and healthy controls to analyze the role of common metabolites, to develop and refine predictive models of X-linked adrenoleukodystrophy and Zellweger syndrome, and to assess analytes with screening utility.

### Patient data

Patient data was collected by the Newborn Screening Center of Shanghai Children’s Hospital in the time period of January 2017 to March 2021 and retrieved from the hospital’s computer system. The dataset contained a total of n = 398 Chinese patients in 4 subsets - a neurological disease subset (DDE) (n = 181) consisting of patients with neurological abnormalities such as developmental delay which did not have a diagnosis of a peroxisomal disorder (non-PD neurological patients); a peroxisomal disorder patients subset (n = 18) consisting of children who had received a diagnosis of X-linked adrenoleukodystrophy (X-ALD, 14 cases, 13 families) and Zellweger syndrome (ZS, 4 cases, 3 families); and healthy controls (n = 199, control) which were selected from the population of patients who had visited for the purpose of routine newborn screening. The following stratifications of the patients were assembled for the subsequent multivariate analysis: (a) a 4-class, consisting of the ZS, X-ALD, DDE and Control classes (b) a 2-class, consisting of ZS and a set combining the X-ALD, DDE, and control patient classes (XDC), and (c) a 2-class, consisting of X-ALD and a set combining ZS, DDE and control patient classes (ZDC).

The patients were of male sex and the average age of the patients was 4.5 years, ranging from age of 1 day to 11 years old. The 18 cases of peroxisomal disorders (14 cases of X-ALD and 4 cases of ZS, all of who were males, with an age range of 2 days to 10 years were diagnosed by high-throughput sequencing technology. In all 14 X-ALD patients hemizygous mutations in the ABCD1 gene were detected, and the 4 cases diagnosed with ZS involved complex mutations in the two genes PEX1 and PEX10. Additional data regarding clinical manifestations and gene mutation results of the 18 peroxisomal disorder patients are presented in Supplementary Table S1.

### Sample collection and mass spectrometry

Collected blood was spotted on S&S 903 (Schleicher & Schuell) filter paper (Merck, Sigma-Aldrich Corp. (St. Louis, MO, USA), dried for 2 h at room temperature and stored at − 20  °C. For each sample, we took a 3 mm diameter dry blood spot of filter paper and placed it in a 96-well microplate (from NeoBase non-derivatized MSMS kit (PerkinElmer Inc, Waltham, MA, USA)) and added 125 µl of the extraction solution (85% methanol, 0.1% oxalic acid, water, and stable isotope internal standard containing ^2^H_3_-Hexacosanoylcarnitine (^2^H_3_-C26) and ^2^H_4_-Hexacosylcholine (^2^H_4_-C26:0 lysophosphatidylcholine, 2H4-C26:0-LPC)). The plate was incubated in a 45  °C airtight manner and oscillated at 600-800 r/min for 30 min. After elution, 100  µl extraction of each sample was transferred into the V-shaped bottom detection plate, and directly tested on the MSMS without chromatographic column. The mobile phase was 84% acetonitrile, 16% water and 0.1% formic acid, and the flow rate of the quaternary pump was set at variable speed as follows: 0.24 mL/min from 0 to 0.15 min, 0.009 mL/min from 0.16 to 1.14 min, 0.6 mL/min from 1.15 to 1.49 min, and 0.12 mL/min from 1.50 to 2.00 min. Each sample took 2 min for analysis, and the injection volume was 20  µl. The MRM parameters were utilized for the relative quantitative determination of the target metabolites as following four VLCAC – eicosanoylcarnitine (C20:0-carnitine), docosanoylcarnitine (C22:0-carnitine), tetracosanoylcarnitine (C24:0-carnitine), hexacosanoylcarnitine (C26:0-carnitine) and the following four LPC- C20:0 lysophosphatidylcholine (C20:0-LPC), C22:0 lysophosphatidylcholine (C22:0-LPC), C24:0 lysophosphatidylcholine (C24:0-LPC) and C26:0 lysophosphatidylcholine (C26:0-LPC). Mass spectrometry detection was performed on a Xevo TQD MS/MS system (Waters, Milford, MA, USA). Sample management was handled by an ACQUITY UPLC I-Class PLUS system (Waters, Milford, MA, USA) comprised of a binary solvent manager (BSM) and a sample manager with Flow-Through Needle (SM-FTN-I).

### Statistical analysis and model development

#### Data processing and feature construction

The concentration levels of the 4 VLCAC features (C20:0-carnitine, C22:0-carnitine, C24:0-carnitine, C26:0-carnitine) and the 4 LPC features (C20:0-LPC, C22:0-LPC, C24:0-LPC, C26:0-LPC) (Table S2), determined using MS/MS for the DDE group (n = 181), the X-ALD (n = 14) group, the ZS (n = 4) group, and the control group (n = 199), were utilized to compute the 4 VLCAC ratio features (C24:0-carnitine/C20:0-carnitine, C24:0-carnitine/C22:0-carnitine, C26:0-carnitine/C20:0-carnitine, C26:0-carnitine/C22:0-carnitine) and the 4 LPC ratio features (C24:0-LPC/C20:0-LPC, C24:0-LPC/C22:0-LPC, C26:0-LPC/C20:0-LPC, C26:0-LPC/C22:0-LPC) (Supplementary Table S2). The four VLCAC and the four LPC metabolites and their 8 ratios were utilized as the 16 features employed in the downstream analysis and model development. The targeted metabolite panel dataset was processed using the R analysis platform, each feature levels were scaled and centered (mean-centering and division by the standard deviation of each feature) [25]. The normalized data was utilized to perform the statistical analysis.

#### Unsupervised analysis (t-SNE and PCA)

Starting with the patient dataset consisting of 398 samples and 16 features, t-Distributed Stochastic Neighbor Embedding (t-SNE) analysis was performed utilizing the R-package Rtsne [26] Complexity was set to number 50 and 1000 iterations were performed. Principal component analysis (PCA) was performed using the R package mixOmics::pca [27].

#### Partial least-squares discrimination analysis (PLS-DA)

Partial least-squares discrimination analysis (PLS-DA) [28] and sparse PLS-DA (sPLS-DA) were performed using the R package mixOmics [27]. Three exploratory PLS-DA models were operated, one model per each stratification: the 4-class ZS/X-ALD/DDE/Control, the 2-class X-ALD/ZDC, and the 2-class ZS/XDC utilizing all available features (n = 16) with a large number (n = 10) of latent components. A 5-fold stratified cross-validation was utilized to assess the number of latent components and classification error metric. VIP values were computed for each feature across the latent components. Then the mixOmics::tune function was utilized to estimate the classification error rate vis-à-vis the number of selected features in the PLS-DA models and to choose a reduced number of features for the final parsimonious models and to re-assess the optimal number of latent components.

Six final sparse PLS-DA models were fitted; two models per each stratification. The performance of each model was assessed by a 5-fold stratified cross-validation repeated 100 times utilizing the mixOmics::perf function, and balanced error rate computation and Area Under Curve(AUC) analysis were performed.

Balanced Error Rate (BER) was computed by first calculating the classification error rate for each class and then the values were averaged to arrive to the balanced error rate. This approach weights all the classes equally regardless of how many samples are in each class. Thus, BER is suitable in situation where there is an unbalanced number of samples in each class - it calculates the average proportion samples that are incorrectly classified for each class, weighted by the available number of samples per class – this approach reduced the bias towards majority classes when performance assessment is performed. A value of BER = [0, 1] indicates a well-performing and significantly accurate model.

## Results and discussion

We analyzed targeted metabolite panel data for n = 398 patients first in an unsupervised manner utilizing t-SNE and PCA, followed by supervised analysis utilizing PLS-DA. Performance of exploratory PLS-DA models for each of the three data set stratifications was assessed to determine a suitable number of latent components and features to retain for sparse PLS-DA models. PLS-DA models with increasing parsimony were fitted in order to establish metabolic signatures of X-linked adrenoleukodystrophy and Zellweger syndrome and assess metabolites significant to model performance.

### Unsupervised analysis: t-SNE and 4-class PCA analysis

**Fig. 1:**
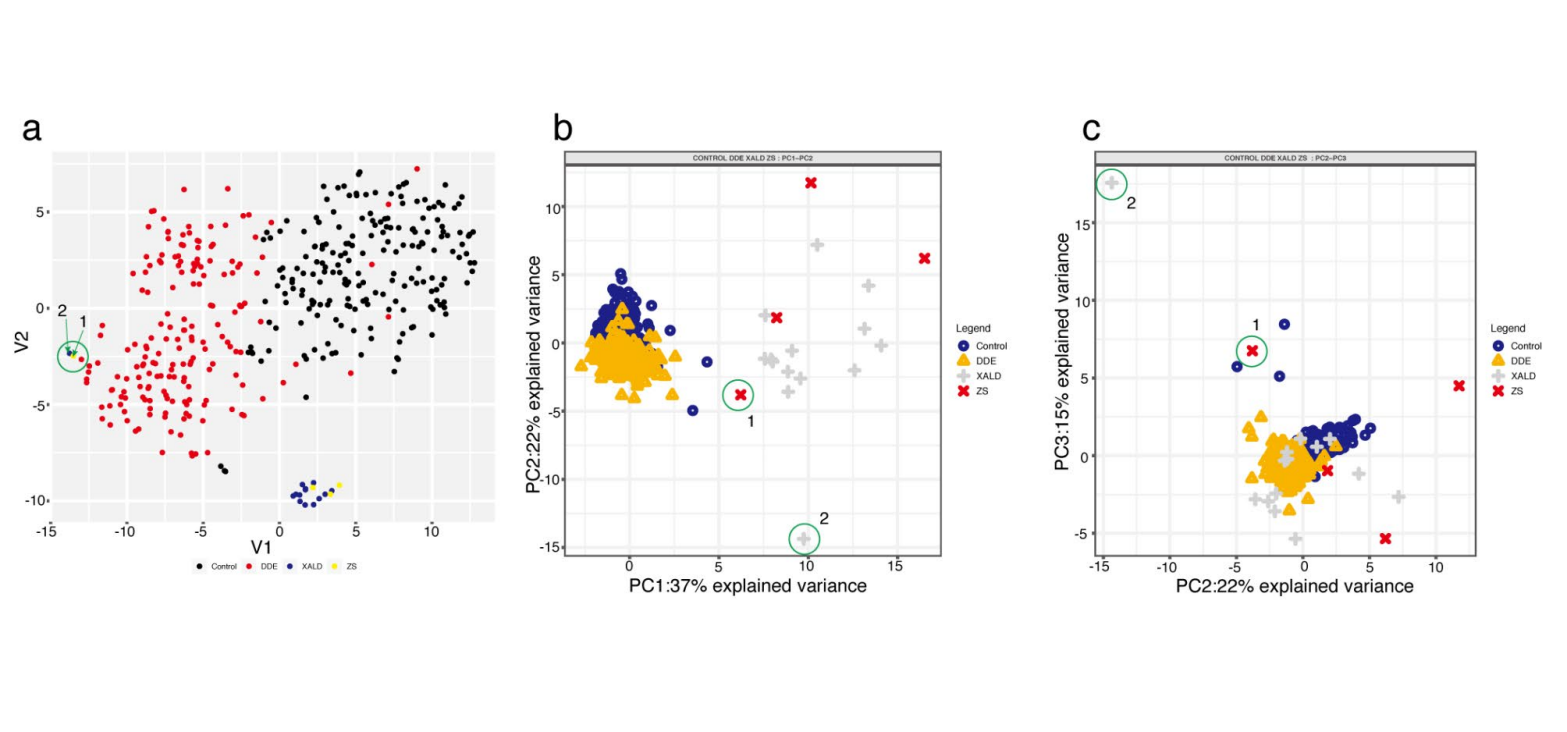
Unsupervised multivariate analysis of patient targeted metabolite panel data. (**a**) t-SNE visualization of 16-features in patient samples for the ZS (yellow), X-ALD (dark blue), DDE (red), and control (black) groups; (**b,c**) PCA analysis of targeted metabolite panel data in healthy controls, non-PD neurological patients (DDE), X-linked adrenoleukodystrophy (X-ALD), and Zellweger syndrome (ZS) patients. Score plots along the 3 principal components are shown.

The targeted metabolite panel dataset in the study was first summarized and explored by an unsupervised step. We calculated median values and concentration ranges, the 1^st^, 50^th^ (median), and 99^th^ percentiles for all 16 features (Supplementary Table S3) followed by the application of dimensionality reduction methods, which allow the data to be visualized in two-dimensions. T-distributed stochastic neighbor embedding (t-SNE) [29] visualization of the 16 metabolite-based features revealed distinct group-specific metabolomics signatures. The control group (n = 199, black dots), ZS group (n = 4, yellow dots), X-ALD group (n = 14, dark blue dots) and DDE group (n = 181, red dots) (Fig. 1, **panel a**) classes show a pattern of separation, with the X-ALD and ZS samples grouping distantly from the DDE and control samples with the notable exception of two samples which appear together near the DDE group.

Next, we performed PCA analysis utilizing the 4 groups (X-ALD, ZS, DDE and control), to examine the intrinsic variation in the patients’ metabolite levels. A PCA model with 3 principal components was fitted. On one hand, we used the method to reduce the dimension of metabolomics data and simplify the data, to visually observe the distribution of samples in the mathematical model space. On the other hand, we used this method to capture the features with the greatest impact and identify the features that are causing the difference in the samples. The DDE and control group do not separate well on the first principal component (PC), whereas the scores plot shows a separation of the ZS and X-ALD samples from the control and DDE groups on PC1 (Fig. 1, panels b, c). Overall, it is immediately apparent that a clustering structure emerges from the observations in the 398 samples.

The proportion of explained variance was determined as 0.37 for PC1, 0.22 for PC2, and 0.15 for PC3, and then cumulatively as 0.37, 0.59, and 0.74 respectively, thus the model explains ~75% of the variation in the lipid metabolite data (Supplementary Fig. 1, Inset). From the scores plots it can be observed that the within class variation of the ZS and X-ALD groups is noticeably higher than the within class variation of DDE and control groups. Features C26:0-carnitine in particular, followed by C24:0-carnitine/C22:0-carnitine, C26:0-carnitine/C20:0-carnitine for the first principal component and C26:0-LPC/C20:0-LPC, C24:0-LPC/C20:0-LPC, C24-0-LPC/C22:0-LPC for the second principal component, appear to have the highest influence on the PCA model as evidenced by the loading plots (**Supplementary Fig. 1, panels a, b**).

Of the two patients that cluster away from the X-ALD and ZS groups, the first patient (ZS) has mutations in PEX10. Metabolite concentration measurements were 0.11 µmol/L (C26:0-carnitine) and 0.46 µmol/L C26:0-LPC). C20:0-carnitine and C22:0-carnitine concentrations for this patient ranked second, and the C24:0-carnitine concentration ranked third in the peroxisomal disorder group. The second patient (X-ALD) has mutations in ABCD1 and had the lowest concentration of 0.07 µmol/L (C26:0-carnitine) and 0.17 µmol /L (C26:0-LPC), and the highest concentrations of C20:0-carnitine, C22:0-carnitine, and C24:0-carnitine amongst the patients from both the ZS and X-ALD groups. In the PCA scores (Fig. 1b, c), the two samples account for the maximum score in PC2 (-14.36 and –3.8) and PC3 (17.56 and 6.75) in the ZS and X-ALD groups.

Overall, the unsupervised analysis results, both by t-SNE and PCA, emphasized the existence of intrinsic group-specific separation within the dataset and the utility to extend the analysis by performing supervised modeling with potential diagnostic application for X-linked adrenoleukodystrophy and Zellweger syndrome patients.

### Supervised classification analysis: PLS-DA and sparse PLS-DA

Next we applied PLS-Discriminant Analysis (PLS-DA) which is a supervised method derived from PLS [30–32] and whose classification power has been widely applied to analyze metabolomics data [33, 34]. The method is readily applicable to modeling in multi-class setting thus allowing us to perform classification analysis where each one of the four classes is considered separately as well as to perform our analysis into the 2-class setting where ZS and X-ALD are the target classes.

#### PLS-DA – exploratory models

**Fig. 2:**
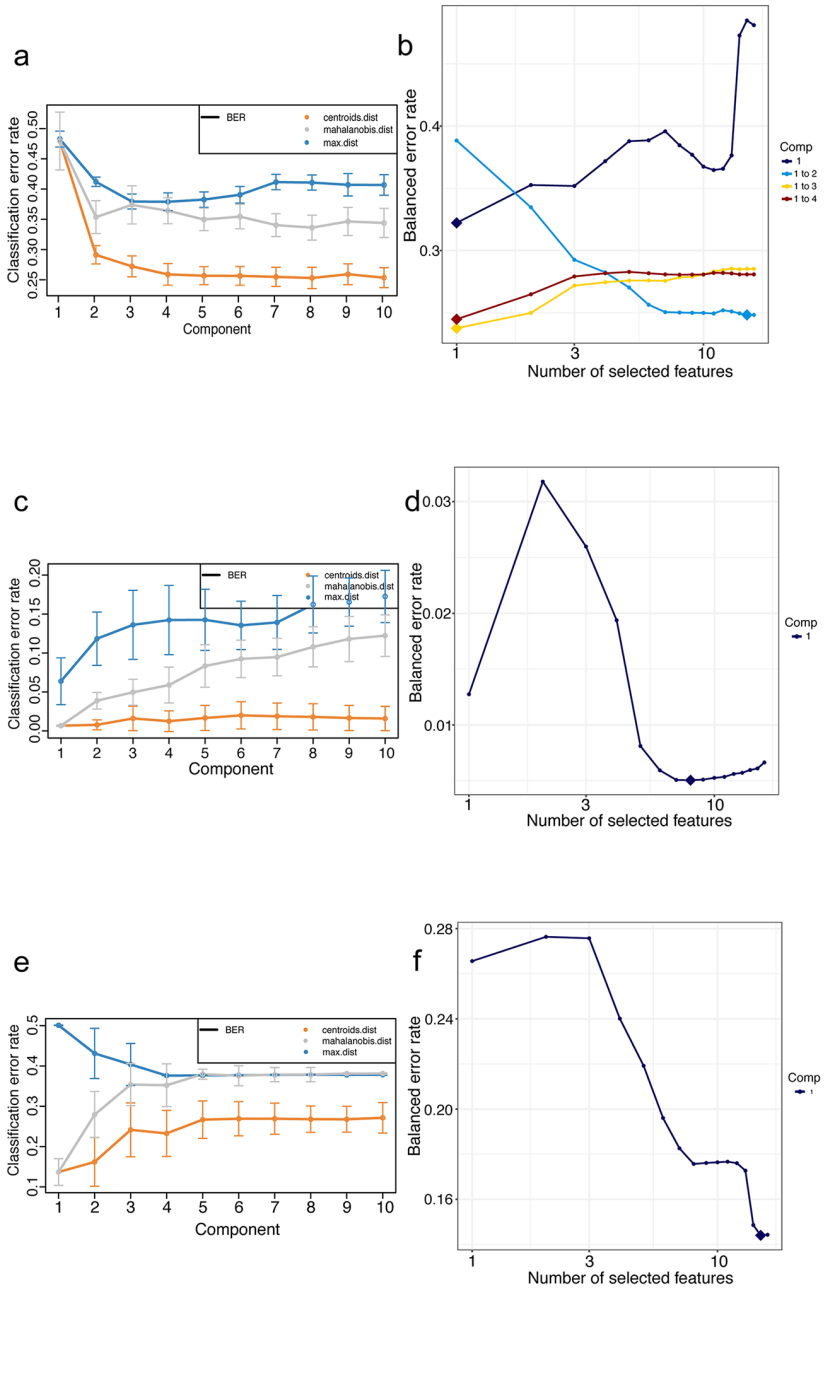
Assessing exploratory PLS-DA model performance and evaluating latent component and features to retain for sparse PLS-DA modeling. **(a, c, e)** Assessing PLS-DA model performance in the 4-class setting (Control vs. DDE vs. X-ALD vs. ZS) and the 2-class setting (X-ALD vs. ZDC; ZS vs. XDC) and selection of distance metric and number of latent components. Repeated stratified cross-validation (100  ×  5–fold CV) is used to evaluate the PLS-DA classification performance (measured by balanced error rate) for each prediction distance (max.dist, centroids.dist, and mahalanobis.dist). The balanced error rate appears to decrease negligibly after four latent components in the 4-class setting, and the balanced error rate reaches minimal value in 2-class setting with 1 latent component. (**b**) Cross-validation and error evaluation of the PLS-DA model in 4-class setting with 4 latent components and all 16 features. Optimal, error minimizing set of features per component are indicated with a diamond. Yellow diamond points to a 3-latent component model with 1, 15, and 1 retained feature(s) per latent components LC1, LC2, and LC3 respectively. (**d**) Cross-validation and error evaluation of the PLS-DA model in X-ALD vs. ZDC 2-class setting with 1 latent component and all 16 features. blue diamond points to a 1 latent component model with 8 retained features. (**f**) Cross-validation and error evaluation of the PLS-DA model in ZS vs. XDC 2-class setting with 1 latent component and all 16 features. Blue diamond points to a 1 latent component model with 15 retained features

A total of three exploratory PLS-DA models including 10 latent components and all 16 features were fitted on the three data partitions (4-class, 2-class X-ALD vs. ZDC, and 2-class ZS vs. XDC). The goal of this step was to evaluate the classification potential of the PLS-DA approach as well as to estimate an appropriate number of latent components and classification distance to be utilized in the subsequent modeling. The number of components to use is a crucial decision and is dictated by the performance of the PLS-DA model – i.e., its ability to correctly classify novel samples. In this step, we performed 5-fold, stratified cross validation with 100 repetitions while evaluation the classification error of the model was done. The repeats were performed to reduce the impact of the randomly allocated folds during each repeat. The following number of latent components emerges as appropriate as the error for each distance metric decreases by very incremental amounts after a subsequent latent component is added and components beyond that are likely to provide negligible returns to the classification performance: n = 4 for the 4-class modeling and n = 1 for the 2-class modeling schemes (Fig. 2, panel a,c,e). Centroids distance and balanced error rate (BER) were employed as distance measure and error rate.

#### PLS-DA models with all features

Next, we analyzed the metabolic differences between the patient groups by using PLS-DA and fitting a model first in the 4-class setting (4LC-FULL) with 4 latent components, utilizing all available 16 features. Supplementary Fig. 2 shows the score plot, VIP plot and ROC curve assessment of the classification performance of the 4LC-FULL model. The X-ALD and ZS classes appears to separate from the control and DDE classes on the first component, whereas the control vs. DDE and X-ALD vs. ZS less so and require additional components to be added for further improvement. **(Supplementary Fig. 2, panel a).** BER was 0.2586180 and AUROC results were 0.9972, 0.9949, 0.9772, 0.9890, for X-ALD vs. Others, ZS vs. Others, DDE vs. Others, and Control vs. Others. **(Supplementary Fig. 2, panel b1-4, Supplementary Table S4**). For the 2-class settings, two models with 1 Latent Component and 16 features were fitted (X-ALD-FULL and ZS-FULL). **(Supplementary Fig. 3, panel a and c**) BER was 0.1315799 and 0.1368718, AUROC was 0.9963 and 0.9898, respectively **Supplementary Table S4**.

The Variable Importance in the Projection (VIP) analyses the importance of the contribution of each feature in explaining the class label through the latent components and summarizes the contribution a variable makes to the model. The VIP score of a variable is calculated as a weighted sum of the squared correlations between the PLS-DA components and the original variable. The weights correspond to the percentage variation explained by the PLS-DA component in the model. An accepted criterion [35] considers features with VIP score > 1 as important for the explanatory power of a model. VIP analysis of the all-features PLS-DA models was applied to screen the features that can best distinguish the target patient groups. Eight features in the 4LC-FULL model were calculated to have VIP > 1: C26:0-carnitine was calculated as the feature with highest VIP score (1.32), C26:0-LPC was ranked third (VIP score = 1.13) (**Supplementary Fig. 2, panel c**). Eight features in the X-ALD-FULL model were calculated to have VIP score > 1: C26:0-carnitine, was calculated as the feature with highest VIP score (VIP score = 1.56), C26:0-LPC was ranked sixth (VIP score = 1.18). (**Supplementary Fig. 3, panel b**). Four features in the ZS-FULL model were calculated to have VIP score > 1: C26:0-LPC/C22:0-LPC was calculated as the feature with highest VIP score (1.89), C26:0-LPC and C26:0-carnitine were ranked 2nd and 4rd (VIP score = 1.64 and 1.29) (**Supplementary Fig. 3, panel d**). These sets represents the features with the highest importance for each modeling scenario and were included as an input set in parsimonious models and represents a potential molecular signature of X-linked adrenoleukodystrophy and Zellweger syndrome patients.

#### Sparse PLS-DA models and targeted metabolite panel signatures of peroxisomal disorders

Metabolomic and targeted metabolite panel signatures of X-linked adrenoleukodystrophy and Zellweger syndrome consisting of a reduced set of the metabolic features explored in this work would have the potential to serve as a targeted diagnostic set to facilitate and expedite diagnosis at patient presentation or pro-actively via newborn screening. Sparse PLS-DA [36, 37] is an extension of canonical PLS-DA which employs a lasso regularization as a variable reduction strategy. We employed this modeling and variable reduction technique, together with a heuristic selection method, with the goal to arrive at such metabolic signatures. The sparse version of PLS-DA is useful to identify discriminative features or the most-predictive features in order to classify the input samples.

Firstly, we evaluated the performance of the three all-features models developed hitherto by performing 5-fold stratified cross validation repeated 1000 times. The BER was calculated per latent component, as features were added iteratively in the modeling process (Fig. 2, **panel b, d, and f**). The evaluation suggested that for the 4-class stratification a 3-latent components sparse PLS-DA model with the following number of features retained by each component would encode a viable candidate – first latent component (LC1) with 1 feature, second latent component (LC2) with 15 features, and third latent component (LC3) with 1 feature (Fig. 2, **panel b, yellow diamond**). For the 2-class stratifications, the results suggested a 1 latent component with 8 and 15 features retained per component, respectively (Fig. 2, **panel d, f, blue diamond**).

The following approaches were taken when developing the parsimonious models. For the 4-class stratification, a sparse PLS-DA model (3LC1-15-1) was fitted with three latent components, with 1, 15, and 1 features per latent component, respectively. For the 2-class data partitions, a sparse PLS-DA model was fitted with one latent component and 8 (X-ALD vs. ZCD) or 15 (ZS vs. XCD) features retained, respectively. Also, for each of the 3 data stratifications a PLS-DA model was fitted employing the features selected by the criteria of VIP > 1 from the all-features models.

In summary, the best performing model measured by the classification error rate (balanced error rate, BER) and AUROC for the 4-class stratification was the sparse PLS-DA model, for the 2-class stratifications were the models utilizing VIP features. While a most parsimonious model of suitable classification performance would satisfy Occam’s razor criteria, selection of which modeling approach to take will be dependent on the particular investigative context and the aims of the practitioner. Further comparative details of each model performance are presented in **Supplementary Table S4**.

**Fig. 3:**
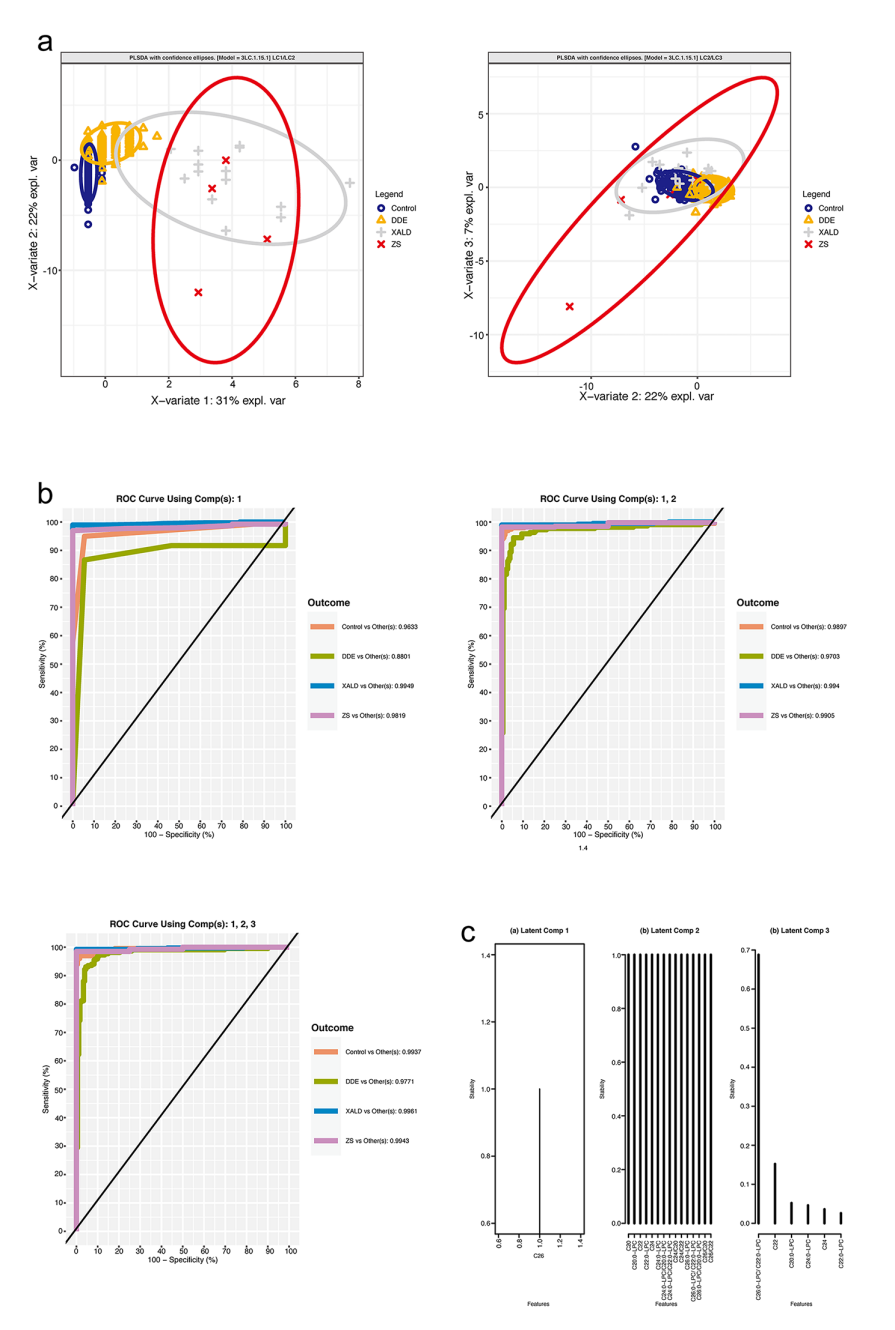
Sparse PLS-DA model 3LC-1-15-1 (4-class setting). (**a1-2**): Sample plots of the targeted metabolite panel data after a parsimonious PLS-DA model was operated on the data, depicting the patient samples with the confidence ellipses for the class labels. (**b1-3**) One-vs.-Others ROC curves assessing the classification performance of the PLS-DA model with 3 latent components and 1, 15, and 1 feature(s) per component (**c**). Feature stability per component evaluation in 5-fold – 100x cross validation

We now provide further details of the fitting and performance of the models. First, a sparse PLS-DA model (3LC-1-15-1) was fitted in the 4-class setting with 3 latent components with 1 feature retained in LC1 and LC3, and 15 features for the latent component LC2. Scores plots (Fig. 3, **panels a, b**) illustrate that the X-ALD and ZS classes are well separated from the control and DDE groups on the first component while similarly the control and DDE group show some overlap. The BER was 0.2433382 (**Supplementary Table S4**). AUROC was 0.9961, 0.9943, 0.9937, 0.9771 for the classification performance of X-ALD vs. Others, ZS vs. Others, Control vs. Others, and DDE vs. Others (Fig. 3, **Panels b1-3, Supplementary Table S4**). The feature(s) selected in the course of the sparse PLS-DA modeling process on LC1 was C26:0-carnitine, thefeatures selected on LC2 and on LC3 are shown and listed on Fig. 3, **Panel c, Supplementary Table S4**. The stability of the feature set was further evaluated in a 5-fold – 100x cross validation where the frequency of features selected was recorded for each repetition. Feature C26:0-carnitine was the only feature selected on LC1 (stability = 1), on LC2 – the features selected had the same stability = 1. On LC3–C26:0-LPC/C22:0-LPC was selected with the highest stability (Fig. 3, **panel c, Supplementary Table S4**).

**Fig. 4:**
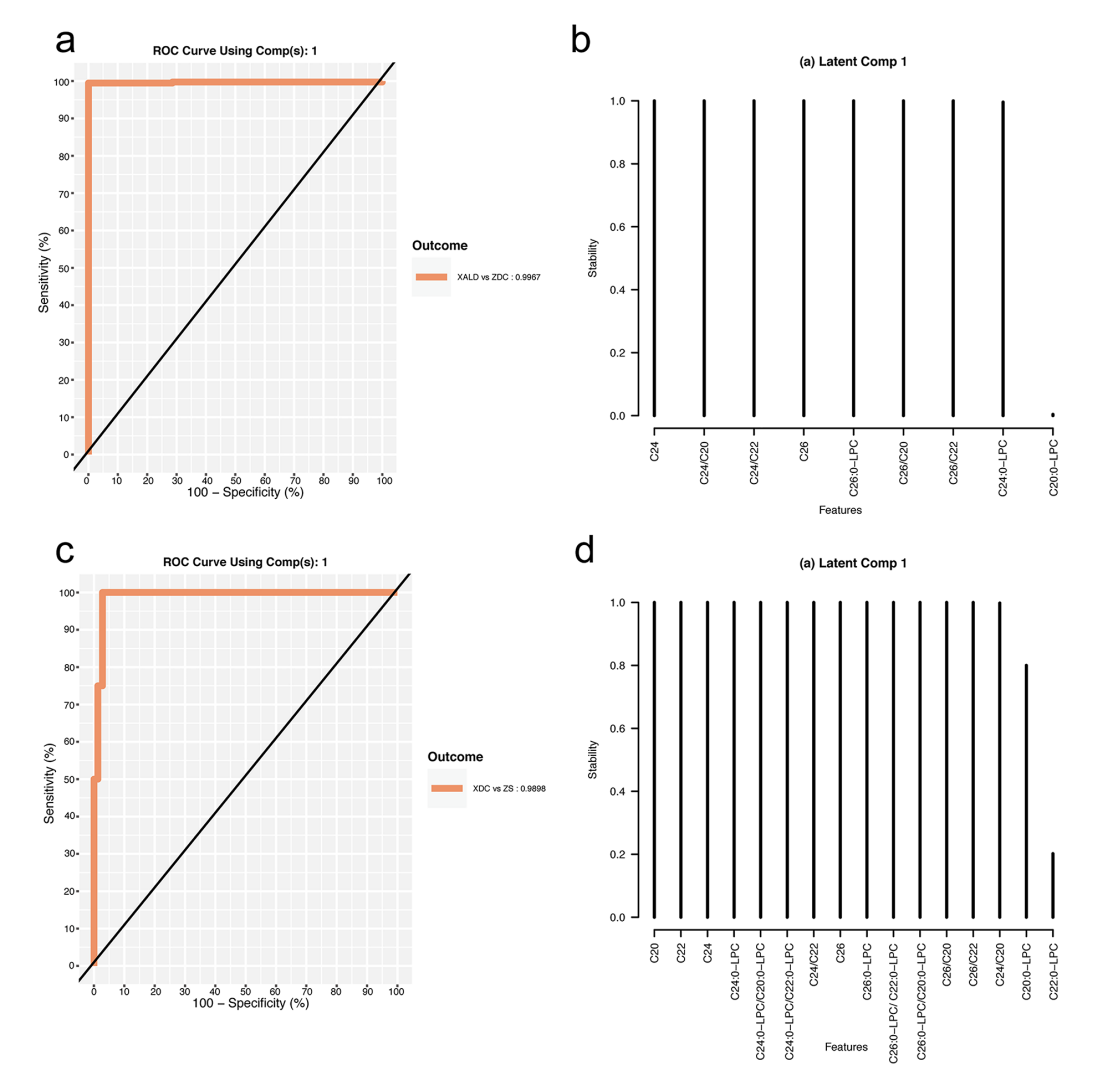
Sparse PLS-DA models in the 2-class settings. (**a**). ROC curve assessing the classification performance of the PLS-DA model with 1 latent components and 8 features in the X-ALD vs. ZDC 2-class setting. (**b**). Feature stability evaluation in 5-fold – 100x cross validation in the X-ALD vs. ZDC 2-class setting. (**c**). ROC curve assessing the classification performance of the sparse PLS-DA model with 1 latent component and 15 features in the ZS vs. XDC 2-class setting. (**d**). Feature stability per in 5-fold – 100x cross validation in the ZS vs. XDC 2-class setting

For the X-ALD vs. ZCD stratification, a sparse PLS-DA model (X-ALD-LC8) was fitted with 1 latent component with 8 features. The BER was 0.005052083 (**Supplementary Table S4**). AUROC was 0.9967 for classification performance X-ALD vs. Others (Fig. 4, **panel a**). The features selected with stability  =  1 in this model were C24-carnitine, C26-carnitine, C24-carnitine/C20-carnitine, C24-carnitine/C22-carnitine, C26-carnitine/C20-carnitine, C26-carnitine/C22-carnitine, and C26:0-LPC. C24:0-LPC was selected with stability slightly < 1 (Fig. 4, **panel b**).

For the ZS vs. XCD stratification, a sparse PLS-DA model (ZS-LC15) was fitted with 1 latent component with 15 features. The BER was 0.1353934 and the AUROC was 0.9898 for classification performance ZS vs. Others (Fig. 4, **panel c, Supplementary Table S4**). 14 of the selected features had stability = 1 (Fig. 4, **panel d**).

These feature set represents a molecular signature that can be used for classification of X-linked adrenoleukodystrophy and Zellweger syndrome patients and distinguish such patients from other neurological disease patients or healthy control individuals. Furthermore, C26:0-carnitine emerges consistently as feature of importance for model classification performance.

**Fig. 5:**
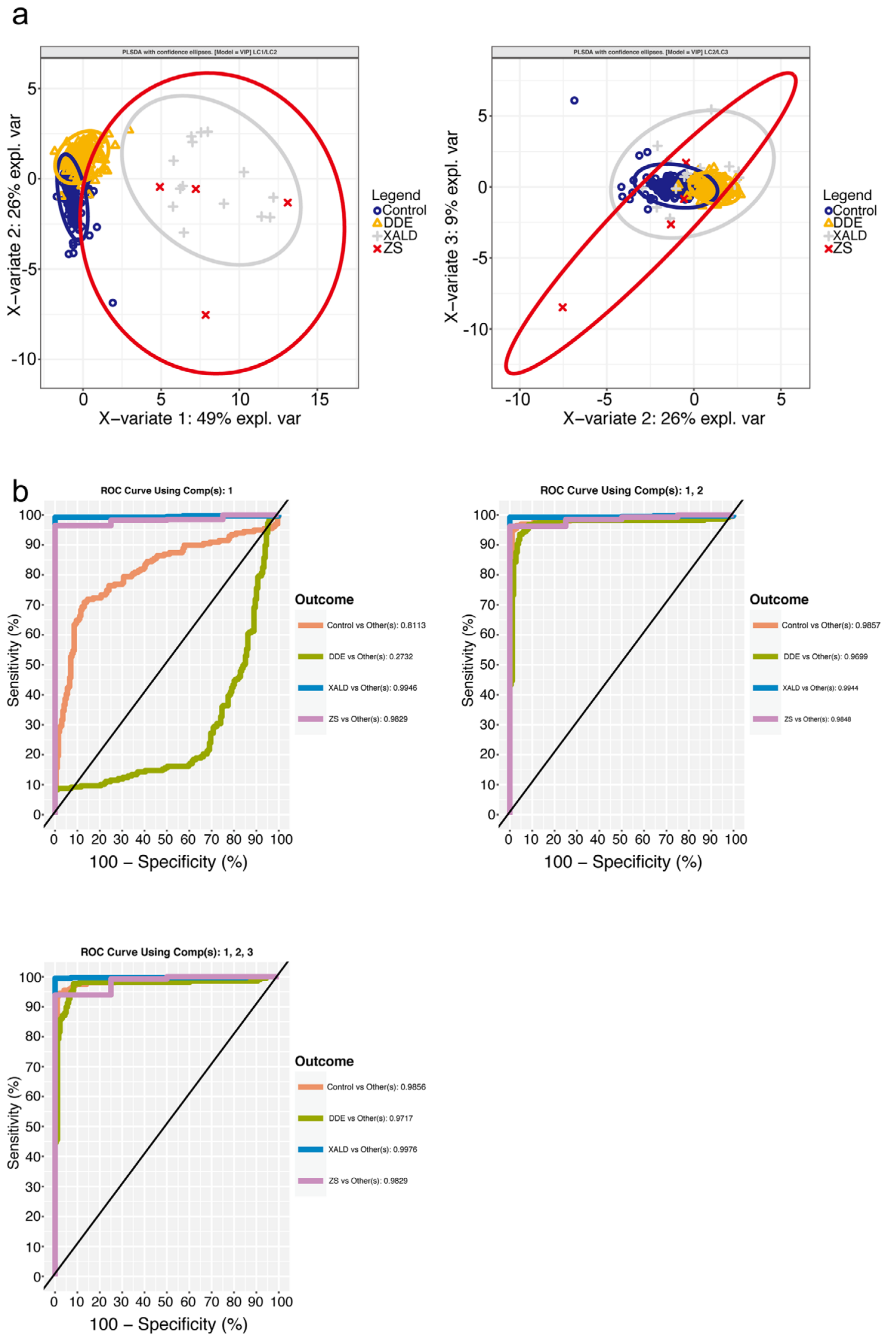
PLS-DA VIP model in the 4-class setting. (**a1-2**) Sample plots after the PLS-DA model with the VIP features was operated on the data, depicting the patient samples with the confidence ellipses for the class labels (**b1-3**) One-vs.-Others ROC curves assessing the classification performance of the PLS-DA model with 3 Latent Components and VIP features

**Fig. 6:**
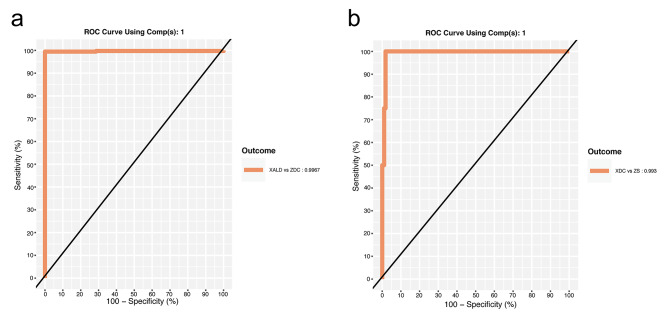
PLS-DA VIP model in 2-class settings. (**a**) ROC curve assessing the classification performance of the PLS-DA model with 1 Latent Components and VIP features in the X-ALD vs. ZDC 2-class setting (**b**) ROC curve assessing the classification performance of the PLS-DA model with 1 Latent Components and VIP features in the ZS vs. XDC 2-class setting

Three VIP models, one model per each data stratification (4C-VIP, X-ALD-VIP, ZS-VIP), were fitted utilizing features set derived from the non-sparse PLS-DA model where VIP analysis yielded VIP score > 1. This set represented the features with the highest importance for the exploratory model and are considered here as a potential molecular signature of X-linked adrenoleukodystrophy and Zellweger syndrome. Performance was again assessed by 5-fold stratified cross validation with 100 repeats and measured by the balanced error rate and AUC. The 4C-VIP model was fitted with 3 latent components and 8 features per component (Fig. 5, **Supplementary Table S4**). Score plot is shown on Fig. 5, **panels a, b**. (Fig. 5**Panel b1-3**) BER was 0.2626092, AUC was 0.9976, 0.9829, 0.9856, 0.9717 for XALD vs. Others, ZS vs. Others, Control vs. Others, DDE vs. Others. The X-ALD-VIP model was fitted utilizing 1 latent component and 8 feature (**Supplementary Table S4**, Fig. 6). For this model BER = 0.004856771 and AUROC = 0.9967. The ZS-VIP model was fitted utilizing 1 latent component and 4 features (Table S4, Fig. 6 panel c, d). For this model BER = 0.132335 and AUROC = 0.993.

### C26:0-carnitine and C26:0-LPC as screening analytes for X-linked adrenoleukodystrophy and Zellweger syndrome

The search for predictive biomarkers has made significant progress yet it is still an ongoing effort as metabolic tests consisting of parsimonious set of biomarkers and measurement have the potential to simplify and shorten the burdensome diagnostic journey of peroxisomal disorders patients. Evidence has been reported for VLCFA accumulation of ABCD1 knockout mice -both C26:0-LPC and C26:0-carnitine levels were highly increased in the brain, spinal cord, and in bloodspots and extended analysis showed likewise elevated levels in X-ALD patient blood spots, thus establishing C26:0-carnitine as a new X-ALD biomarker in humans (and mice) [38]. Further work evaluated C26:0-LPC and C26:0-carnitine as screening markers for X-ALD [39] and Zellweger spectrum disorders [40], and elevated levels of C26:0-carnitine have been observed in clinical samples of Zellweger spectrum disorders patients [41]. In 2017, Huffnagel et al. [39], when comparing the utility of analyzing C26:0 carnitine vis-à-vis C26:0-LPC in DBS showed that C26:0-LPC is elevated in all and C26:0-carnitine was elevated in 83% of the examined X-ALD newborns. The authors concluded the superiority of C26:0-LPC compared to C26:0-carnitine for the task of screening of X-ALD in newborn children. In 2020, Jaspers and coworkers [42] compared the screening performance of C26:0-LPC and VLCFA analysis in plasma, concluding a superiority of C26:0-LPC and recommending the utilization of C26:0-LPC analysis in in the diagnosis of peroxisomal disorders. In 2021, Natarajan and coworkers [43] measured C24:0-carnitine and C26:0-carnitine in DBS in an X-ALD cohort of Indian patients and concluded their ability less reliable in comparison with that of C24:0-LPC and C26:0-LPC in screening X-ALD when a cut-off value is applied.

The values measured for C26:0-carnitine and C26:0-LPC in the Chinese pediatric dataset utilized in this work do not allow either one of these analytes to be used to apply a cutoff in separating the four patient classes - non-PD neurological patients, X-linked adrenoleukodystrophy, Zellweger syndrome, and healthy controls from each other or X-linked adrenoleukodystrophy from Zellweger syndrome patients. Furthermore, C26:0-LPC in this study population had minimal ability to separate X-ALD and ZS from the control group (**Supplementary Fig. 4**). The multivariate modeling and classification work performed in our study highlights C26:0-carnitine as a potentially clinically useful analyte, an important feature in multivariate setting, with further support of additional metabolic features. Our paper strengthens the evidence for C26:0-carnitine, such as the one presented by van de Beek et al. (2016) [38] and Tian et all (2020) [22], as a potential analyte with screening utility for X-linked adrenoleukodystrophy and Zellweger syndrome and that a reduced features set and multivariate modeling technique such as sparse PLS-DA can achieve suitable performance in the classification of X-linked adrenoleukodystrophy and Zellweger syndrome patients and neurological disorder patients. The assertion that C26:0-carnitine in DBS is a preferable and more accurate analyte to be utilized as a primary biomarker for peroxisomal disorders may be premature, yet it certainly merits further investigation and clinical validation particularly in the context of advanced discriminative models in lieu of straight cutoffs and in light of the fact that C26:0-carnitine can be introduced with ease as an additional analyte in existing high throughput acylcarnitine and amino acid testing. Furthermore, ethnicity is a contributing factor in the variation of newborn screening. The dataset examined in this work is sourced from the screening program of a primary Chinese hospital in Shanghai and consists solely of Chinese patients, thus the results obtained may support the notion of a demographic effect (Asian vs. Caucasian patients) while showing that C26:0-carnitine can be a useful analyte for newborn screening of patients of Chinese ethnicity, which merits further investigations and consideration.

## Conclusions

We have uncovered metabolic profiles of peroxisome disorders using MS/MS and multivariate statistical analysis, reporting relevant biological information to distinguish between healthy controls, non-PD neurological patients, X-linked adrenoleukodystrophy, and Zellweger syndrome patients. The VLCAC and LPC metabolic profiles showed a highly significant altered metabolic state in X-linked adrenoleukodystrophy and Zellweger syndrome patients. PLS-DA classification models with reduced feature set were developed achieving excellent classification power and AUROC. Our study demonstrated metabolic differences between healthy controls, non-PD neurological patients and X-linked adrenoleukodystrophy and Zellweger syndrome patients, and showed the potential utility of C26:0-carnitine as a relevant screening analyte for Chinese patients in the context of a discriminative model predictive of disease state.

## Electronic supplementary material

Below is the link to the electronic supplementary material.

Supplementary Material 1

Supplementary Material 2

## Data Availability

The data presented in this study are available on request. The data are not publicly available due to privacy restrictions.

## Abbreviations

X-ALD: X-linked Adrenoleukodystrophy
ZS: Zellweger Syndrome
VLCFA: Very long chain fatty acids
VLCAC: Very long chain acylcarnitines
LPC: Lysophosphatidylcholine
C20:0-carnitine: Eicosanoylcarnitine
C22:0-carnitine: Docosanoylcarnitine
C24:0-carnitine: Tetracosanoylcarnitine
C26:0-carnitine: Hexacosanoylcarnitine
C20:0-LPC: C20:0-lysophosphatidylcholine
C22:0-LPC: C22:0-lysophosphatidylcholine
C24:0-LPC: C24:0-lysophosphatidylcholine
C26:0-LPC: C26:0-lysophosphatidylcholine
DDE: non-PD neurological patients subset
t-SNE: t-Distributed Stochastic Neighbor Embedding
PCA: Principal Component Analysis
PLS-DA: Partial Least-Squares Discrimination Analysis
sPLS-DA: Sparse PLS-DA
BER: Balanced error rate
VIP: Variable Importance in the Projection
AUC: Area Under Curve
