# Correction: Multivariate analysis and model building for classifying patients in the peroxisomal disorders X-linked adrenoleukodystrophy and Zellweger syndrome in Chinese pediatric patients

**DOI:** 10.1186/s13023-023-02752-z

**Published:** 2023-06-15

**Authors:** Zhixing Zhu, Georgi Z. Genchev, Yanmin Wang, Wei Ji, Xiaofen Zhang, Hui Lu, Sira Sriswasdi, Guoli Tian

**Affiliations:** 1 Shanghai Engineering Research Center for Big Data in Pediatric Precision Medicine; Center for Biomedical Informatics, Shanghai Children’s Hospital; School of Medicine, Shanghai Jiao Tong University, Shanghai, China; 2 Center of Excellence in Computational Molecular Biology, Faculty of Medicine, Chulalongkorn University, Bangkok, Thailand; 3 Newborn Screening Center, Shanghai Children’s Hospital, School of Medicine, Shanghai Jiao Tong University, Shanghai, China; 4 SJTU-Yale Joint Center for Biostatistics, Department of Bioinformatics and Biostatistics, Shanghai Jiao Tong University, Shanghai, China

**Correction: Orphanet Journal of Rare Diseases (2023) 18:102** **https://doi.org/10.1186/s13023-023-02673-x**

Following publication of the original article [1], we have been notified that the affiliation 5 (Key Laboratory of Digital Technology in Medical Diagnostics of Zhejiang Province, Zhejiang, China) should not be assigned to author Guoli Tian. Also, statement of equal contribution is missing. It should be as follows:

Guoli Tian^3*^

Zhixing Zhu and Georgi Z. Genchev are first co-authors with equal contribution.
