# Supplementary material for: Multivariate analysis and model building for classifying patients in the peroxisomal disorders X-linked adrenoleukodystrophy and Zellweger syndrome in Chinese pediatric patients
Source: Orphanet J Rare Dis. 2023 May 2;18:102. doi: 10.1186/s13023-023-02673-x (PMC10186734; doi:10.1186/s13023-023-02673-x)
Supplement: Supplementary file 1 — Supplementary Material 1 [file 13023_2023_2673_MOESM1_ESM.docx]

**
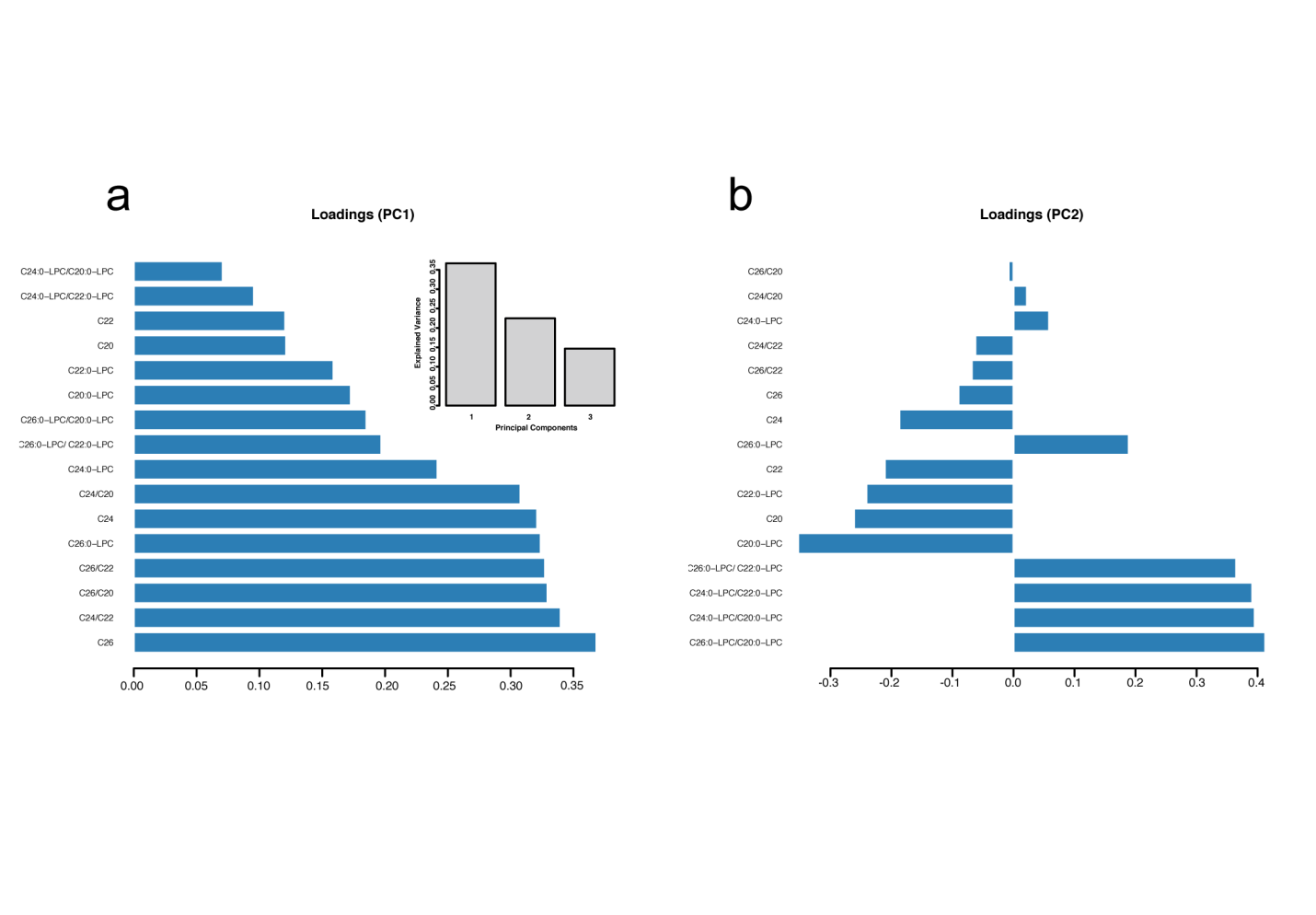
**

**Supplementary Figure 1:**

**(a, b):** Loading plots from the PCA applied to the metabolomics data. Loading weights are ranked from the most important (bottom) to the least important (top) on PC1 and PC2. **Inset**: Proportion of explained variance per the 3 principal components.


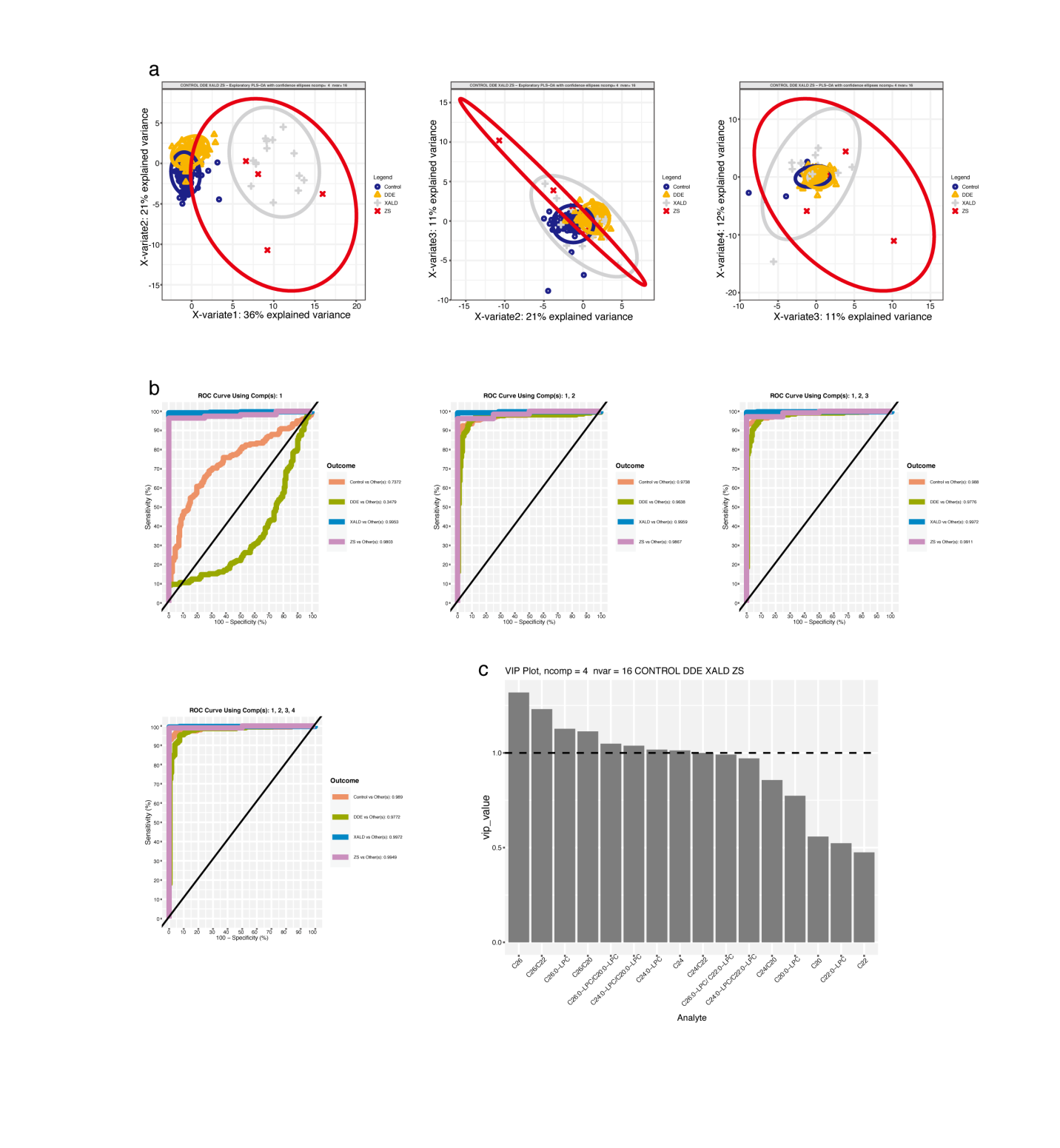


**Supplementary Figure 2:**

PLS-DA model in the 4-class setting with 4 latent components and 16 features **(a1-3):** Sample plots after a PLS-DA model was operated on the patient metabolomics data. Confidence ellipses are shown for the different classes. **(b1-4)** One-vs.-Others ROC curves assessing the classification performance of the PLS-DA model **(c)** bar plot of the variables ranked by VIP value. The VIP value is averaged across the 4 latent components

**
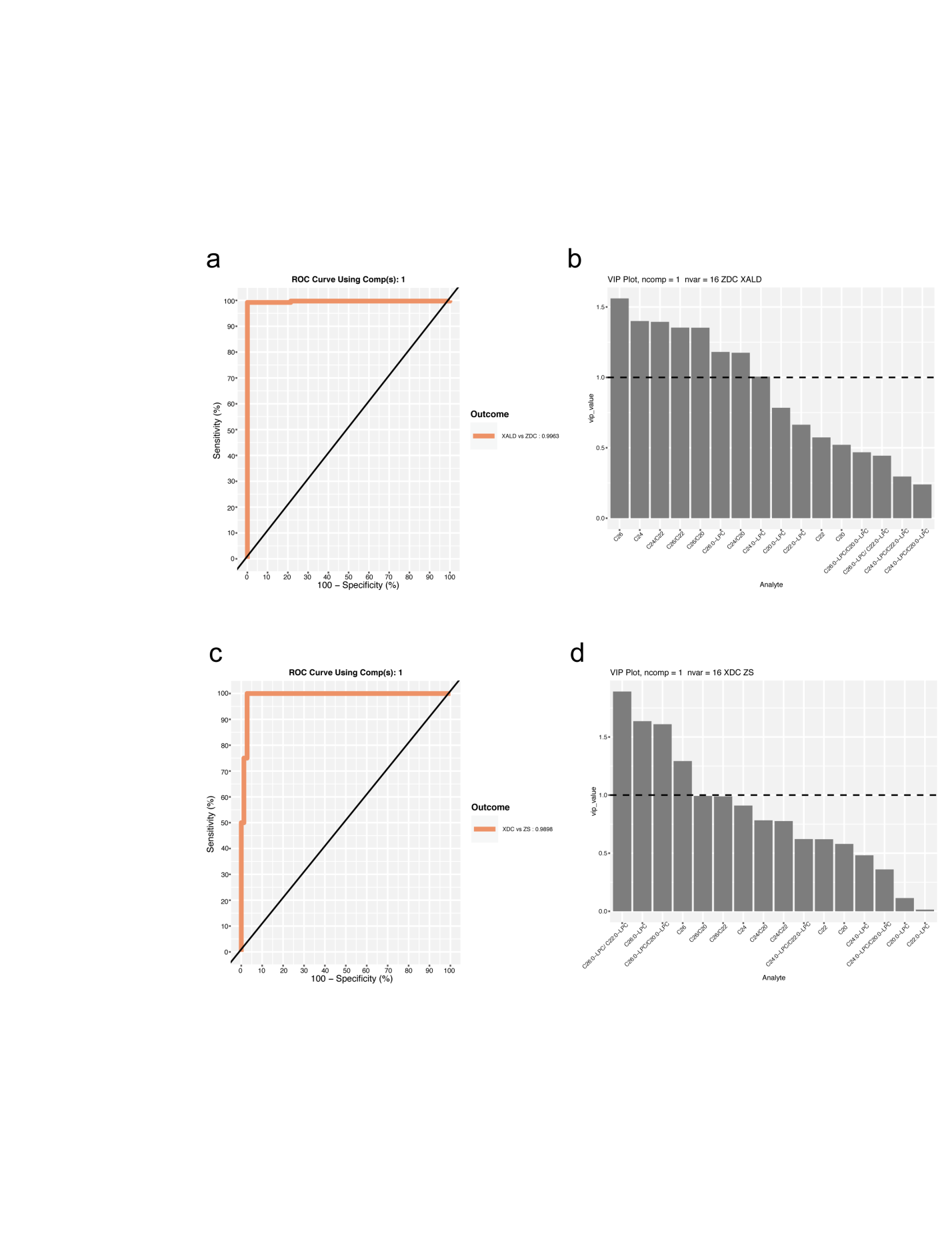
**

**Supplementary Figure 3:**

PLS-DA model in the 2-class setting with 1 latent components and 16 features. **(a)** ROC curves assessing the classification performance of the PLS-DA model in X-ALD vs ZDC **(b)** bar plot of the variables ranked by VIP value in X-ALD vs. ZDC. **(c)** ROC curves assessing the classification performance of the PLS-DA model in ZS vs. XDC. **(d)** bar plot of the variables ranked by VIP value in ZS vs. XDC. Abbreviations: (Zellweger syndrome (ZS), healthy controls (Control), X-linked adrenoleukodystrophy (X-ALD), non-PD neurological patients (DDE), combined set of ZS, DDE and Controls (ZCD), combined set of X-ALD, DDE and Controls (XCD)


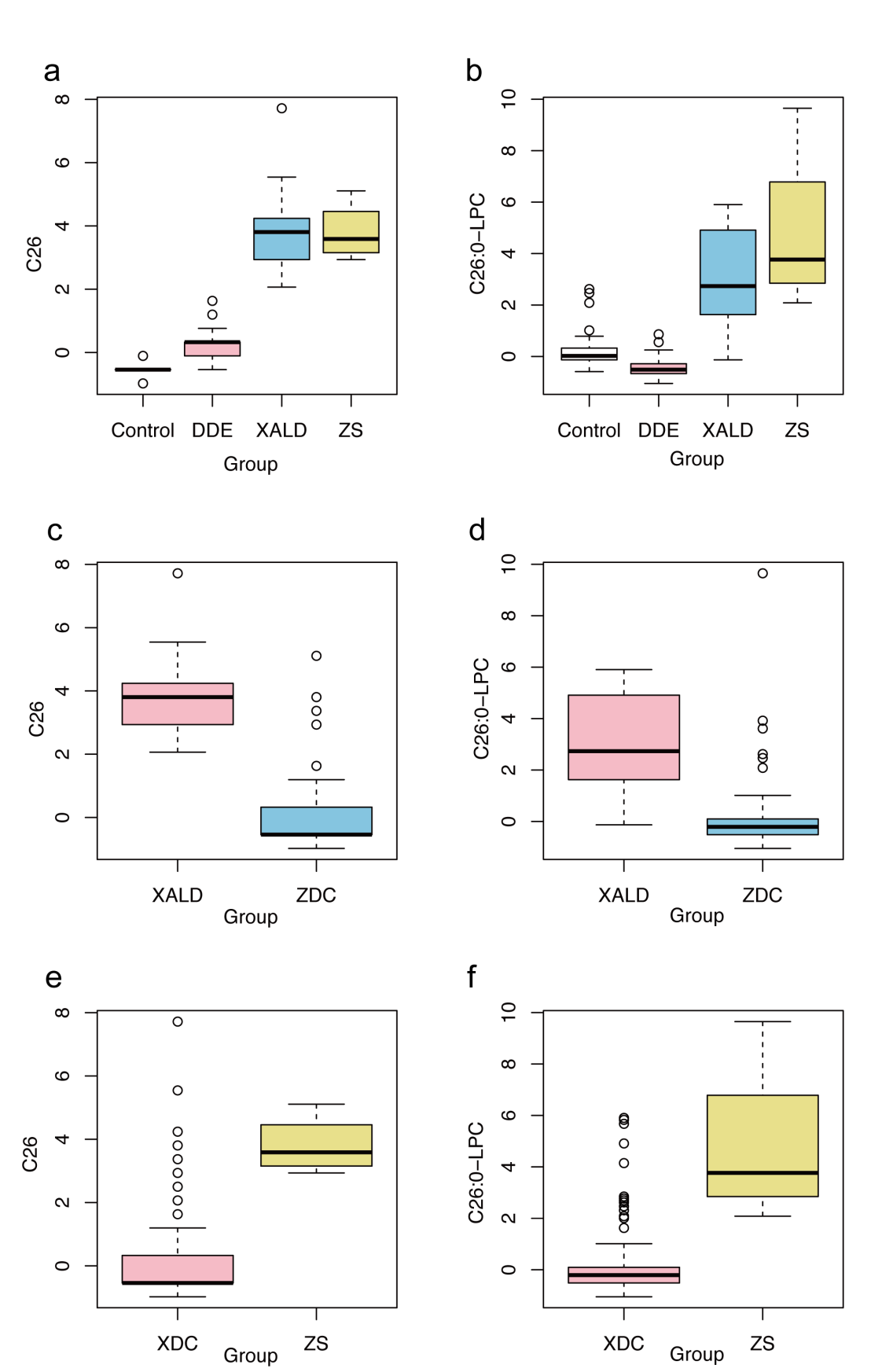


**Supplementary Figure 4:**

Bar plot of C26:0-carnitine **(a, c, e)** and C26:0-LPC **( b, d, f)** metabolite levels for the 4-class(Control, DDE, X-ALD, ZS) setting and the 2-class setting (X-ALD vs. ZDC, ZS vs. XDC). Abbreviations: (Zellweger syndrome (ZS), healthy controls (Control), X-linked adrenoleukodystrophy (X-ALD), non-PD neurological patients (DDE), combined set of ZS, DDE and Controls (ZCD), combined set of X-ALD, DDE and Controls (XCD),
