# Supplementary material for: Multivariate analysis and model building for classifying patients in the peroxisomal disorders X-linked adrenoleukodystrophy and Zellweger syndrome in Chinese pediatric patients
Source: Orphanet J Rare Dis. 2023 May 2;18:102. doi: 10.1186/s13023-023-02673-x (PMC10186734; doi:10.1186/s13023-023-02673-x)
Supplement: Supplementary file 2 — Supplementary Material 2 [file 13023_2023_2673_MOESM2_ESM.docx]

**Table S1 Clinical manifestations and gene mutation results of 18 patients**

| Samples | Age | Pathogenic gene | Genetic pattern | Pathogenic site | | Clinical features | MRI |
| --- | --- | --- | --- | --- | --- | --- | --- |
|  |  |  |  | cDNA base-change | Amino acid change |  |  |
| 1 | 8.3y | ABCD1 | XR | c.1552C>T | p.Arg518Trp | hypopsia | large symmetrical patches of abnormal signals in the white matter around the posterior horn of bilateral ventricles and the trigone |
| 2 | 5.2y | ABCD1 | XR | c.1850G>A | P.Arg617His | hypopsia | Abnormal signal in pons and bilateral paraventricular posterior horn |
| 3 | 5.5y | ABCD1 | XR | c.818C>T | p.Ala273Val | Mental delay | A few abnormal signals near bilateral ventricles |
| 4 | 6.0y | ABCD1 | XR | c.1415_1416delAG | p.Gln472Argfs*83 | hypopsia、intellectual disability | Abnormal signal in white matter area beside posterior horn of bilateral lateral ventricles |
| 5 | 8.6y | ABCD1 | XR | c.1202G>A | p.Arg401Gln | hypopsia、Unsteady gait with binaural hearing loss | Abnormal signals beside bilateral lateral ventricles |
| 6# | 2.5y | ABCD1 | XR | c.310C>T | p.Arg104Cys | Mental delay | Local white matter changes in bilateral posterior horn of lateral ventricles and bilateral frontal parietal lobes |
| 7# | 7.5y | ABCD1 | XR | c.310C>T | p.Arg104Cys | Cognitive and motor regression | Local white matter changes in bilateral posterior horn of lateral ventricles and bilateral frontal parietal lobes |
| 8 | 8.2y | ABCD1 | XR | c.1553G>A | p.Arg518Gln | Intellectual regression | Demyelination of white matter in the parahorn of bilateral ventricles |
| 9 | 6.2y | ABCD1 | XR | c.1553G>A | p.Arg518Gln | Hearing loss | Multiple symmetrical abnormal signals in the brain |
| 10 | 10y | ABCD1 | XR | c.1892G>A | p.Cys631Tyr | Learning Disability、Attention loss | Abnormal signal in white matter area beside posterior horn of bilateral lateral ventricles |
| 11 | 5d | ABCD1 | XR | c.1876G>A | p.Ala626Thr | Hyperbilirubin encephalopathy | —— |
| 12 | 2.2y | ABCD1 | XR | c.1028G>A | p.Gly343Vla | Persistent seizures、Mental delay | Abnormal symmetrical signal in dentate nucleus of cerebellar hemisphere  Local white matter changes near the posterior horn of bilateral lateral ventricles |
| 13 | 10m | ABCD1 | XR | c.395G>A | p.Trp132* | Persistent seizures | Local white matter changes near the posterior horn of bilateral lateral ventricles  Incomplete myelination of white matter |
| 14 | 5.8y | ABCD1 | XR | c.629A>T | p.His210Leu | Hemiplegia, unable to walk and speak | Large symmetric patchy abnormal signals near bilateral ventricles |
| 15# | 2.2y | PEX1 | AR | c.2966T>C | p.Ile989Thr | Growth delay、Unstable gait | Abnormal signals beside bilateral lateral ventricles |
|  |  |  |  | c.1246_1247delGA | p.Asp416* |  |  |
| 16# | 4.6y | PEX1 | AR | c.2966T>C | p.Ile989Thr | Hearing loss、Unsteady gait、limb asthenia | Intracranial broadly symmetric abnormal signal |
|  |  |  |  | c.1246_1247delGA | p.Asp416* |  |  |
| 17 | 2d | PEX1 | AR | c.2050C>T | p.Gln684* | Hypotonia、Special face | —— |
|  |  |  |  | c.782_783delAA | p.Gln261Argfs*8 |  |  |
| 18 | 4.7y | PEX10 | AR | c.284T>C | p.Leu95Pro | Indifferent and irritable | Symmetric abnormal signals in brainstem、Bilateral cerebellar hemispheres、  bilateral temporo occipital parietal lobe and bilateral thalamic regions |
|  |  |  |  | c.284T>C | p.Leu95Pro |  |  |

"——" ：No data

Inheritance mode: XR (X chromosome recessive inheritance), AR (autosomal recessive inheritance)

#: from the same family

**Table S2: (Features) 16 model features representing VLCAC and LPC levels in patient samples.**

| **VLCAC features** | **LPC feature** | **VLCAC ratio features** | **LPC ratio features** |
| --- | --- | --- | --- |
| C20:0-carnitine | C20:0-LPC | C24:0-carnitine /C20:0-carnitine | C24:0-LPC/C20:0-LPC |
| C22:0-carnitine | C22:0-LPC | C24:0-carnitine /C22:0-carnitine | C24:0-LPC/C22:0-LPC |
| C24:0-carnitine | C24:0-LPC | C26:0-carnitine /C20:0-carnitine | C26:0-LPC/C20:0-LPC |
| C26:0-carnitine | C26:0-LPC | C26:0-carnitine /C22:0-carnitine | C26:0-LPC/C22:0-LPC |

**Table S3: Descriptive Statistics**

| Features | Control group (n=199)  Median (range) | DDE group (n=181)  Median (range) | X-ALD group (n=14)  Median (range) | ZS group (n=4)  Median (range) |
| --- | --- | --- | --- | --- |
| C20:0-carnitine | 0.01(0.01-0.03) | 0.02(0.01-0.03) | 0.02(0.01-0.15) | 0.025(0.01-0.07) |
| C22:0-carnitine | 0.01(0.01-0.01) | 0.01(0.01-0.02) | 0.01(0.01-0.13) | 0.015(0.01-0.06) |
| C24:0-carnitine | 0.02(0.01-0.04) | 0.02(0.01-0.06) | 0.1(0.06-0.33) | 0.095(0.04-0.14) |
| C26:0-carnitine | 0.01(0.01-0.02) | 0.03(0.01-0.05) | 0.11(0.07-0.20) | 0.11(0.09-0.14) |
| C20:0-LPC | 0.18(0.07-0.53) | 0.24(0.1-0.47) | 0.4(0.13-0.71) | 0.24(0.14-0.44) |
| C22:0-LPC | 0.14(0.09-0.52) | 0.15(0.07-0.36) | 0.28(0.09-0.48) | 0.16(0.11-0.25) |
| C24:0-LPC | 0.38(0.23-1.15) | 0.25(0.11-0.51) | 0.83(0.40-1.39) | 0.62(0.46-0.73) |
| C26:0-LPC | 0.19(0.12-0.46) | 0.12(0.05-0.23) | 0.55(0.18-0.96) | 0.68(0.47-1.43) |
| C24:0-carnitine /C20:0-carnitine | 2(0.50-4) | 1.5(0.5-4) | 5(2.25-9.74) | 2.75(1.35-11.75) |
| C24:0-carnitine /C22:0-carnitine | 2(1-4) | 2(1-5) | 7.5(2.68-11) | 4.67(2.01-11.85) |
| C26:0-carnitine /C20:0-carnitine | 1(0.25-2) | 1.5(0.5-4.2) | 6.33(0.88- 11) | 4(1.61-13.73) |
| C26:0-carnitine /C22:0-carnitine | 1(1-2) | 2(1-5) | 9(0.55-14.61) | 7.25(1.91-13.88) |
| C24:0-LPC/C20:0-LPC | 2.10(1.15-4.73) | 1(0.43-2.64) | 2.06(0.96-4.23) | 2.42(1.68-4.10) |
| C24:0-LPC/C22:0-LPC | 2.62(1.56-4.70) | 1.55(0.72-3.29) | 2.43(1.47-6.13) | 3.83(2.94-4.81) |
| C26:0-LPC/C20:0-LPC | 1.05(0.52-2.67) | 0.52(0.18-1.32) | 1.34(0.28-4.03) | 3.64(1.08-7.96) |
| C26:0-LPC/C22:0-LPC | 1.33(0.68-2.37) | 8(0.28-1.95) | 1.82(0.38-5.92) | 4.65(1.89-12.96) |

Median value and range of the features concentration in the control, DDE, X-ALD and ZS groups. Range is expressed in between the 1^st^ and the 99^th^ percentile. * Data shown: median (range). Abbreviations: Zellweger syndrome (ZS), healthy controls (Control), X-linked adrenoleukodystrophy (X-ALD), non-PD neurological patients (DDE), combined population of ZS, DDE and Controls (ZCD), combined population of X-ALD, DDE and Controls (XCD)

**Table S4: PLS-DA, sparse PLS-DA, and PLS-DA (VIP features) model performance comparison**

**a) Classification errors**

| **Classes** | **Model** | **4LC-full** | **3LC-1-15-1** | **VIP** |
| --- | --- | --- | --- | --- |
| Control/DDE/X-ALD/ZS | Classification error | BER | BER | BER |
|  | LC:1 | 0.4852636 | 0.3254334 | 0.4621403 |
|  | LC:2 | 0.2915735 | 0.2501824 | 0.2947068 |
|  | LC:3 | 0.2698692 | 0.2433382 | 0.2626092 |
|  | LC:4 | 0.2586180 | / | / |
| X-ALD/ZDC | **Model** | **XALD-full** | **LC-8** | **VIP-8** |
|  | Classification error | BER | BER | BER |
|  | LC:1 | 0.1315799 | 0.005052083 | 0.004856771 |
| ZS/XDC | **Model** | **ZS-full** | **LC-15** | **VIP-4** |
|  | Classification error | BER | BER | BER |
|  | LC:1 | 0.1368718 | 0.1353934 | 0.132335 |

**b) AUROC**

| **Model** | **4LC-full** | | | | | **3LC-1-15-1** | | | **VIP** | | |
| --- | --- | --- | --- | --- | --- | --- | --- | --- | --- | --- | --- |
| **AUROC** | LC1 | LC2 | LC3 | | LC4 | LC1 | LC2 | LC3 | LC1 | LC2 | LC3 |
| Control vs. Others | 0.7372 | 0.9738 | 0.9880 | | 0.9890 | 0.9633 | 0.9897 | 0.9937 | 0.8113 | 0.9857 | 0.9856 |
| DDE vs. Others | 0.3479 | 0.9638 | 0.9776 | | 0.9772 | 0.8801 | 0.9703 | 0.9771 | 0.2732 | 0.9699 | 0.9717 |
| XALD vs. Others | 0.9953 | 0.9959 | 0.9972 | | 0.9972 | 0.9949 | 0.9940 | 0.9961 | 0.9946 | 0.9944 | 0.9976 |
| ZS vs Others | 0.9803 | 0.9867 | 0.9911 | | 0.9949 | 0.9819 | 0.9905 | 0.9943 | 0.9829 | 0.9848 | 0.9829 |
| **Model** | **LC.XALD-full** | | | **LC-8** | | | | | **VIP-8** | | |
| **AUROC** | LC1 | | | LC1 | | | | | LC1 | | |
| X-ALD vs. ZDC | 0.9963 | | | 0.9967 | | | | | 0.9967 | | |
| **Model** | **LC.ZS-full** | | | **LC-15** | | | | | **VIP-4** | | |
| **AUROC** | LC1 | | | LC1 | | | | | LC1 | | |
| ZS vs. XDC | 0.9898 | | | 0.9898 | | | | | 0.993 | | |

**c) Features for each latent component**

| **Classes** | **Model** | **3LC-1-15-1** | | | **VIP** | | |
| --- | --- | --- | --- | --- | --- | --- | --- |
| Control/DDE/X-ALD/ZS | LC | LC1 | LC2 | LC3 | LC1 | LC2 | LC3 |
|  | Features | C26:0-carnitine | C20:0-carnitine  C22:0-carnitine  C24:0-carnitine  C24:0-carnitine/C22:0-carnitine  C24:0-carnitine /C20:0-carnitine  C26:0-carnitine  /C22:0-carnitine  C26:0-carnitine  /C20:0-carnitine  C20:0-LPC  C22:0-LPC  C24:0-LPC  C26:0-LPC  C26:0-LPC/C22:0-LPC  C26:0-LPC/C20:0-LPC  C24:0-LPC/C20:0-LPC  C24:0-LPC/C22:0-LPC | C26:0-LPC/C22:0-LPC | C24:0-carnitine  C26:0-carnitine  C26:0-carnitine/C20:0-carnitine  C26:0-carnitine/C22:0-carnitine  C24:0-LPC  C26:0-LPC  C26:0-LPC/ C20:0-LPC  C24:0-LPC/ C20:0-LPC | C24:0-carnitine  C26:0-carnitine  C26:0-carnitine /C20:0-carnitine  C26:0-carnitine/ C22:0-carnitine  C24:0-LPC  C26:0-LPC  C26:0-LPC/C20:0-LPC  C24:0-LPC/C20:0-LPC | C24-carnitine  C26-carnitine  C26-carnitine /C20-carnitine  C26-carnitine /C22-carnitine  C24:0-LPC  C26:0-LPC  C26:0-LPC/ C20:0-LPC  C24:0-LPC/ C20:0-LPC |
| XALD/ZDC | **Model** | **LC-8** | | | **VIP-8** | | |
|  | LC | LC1 | | | LC1 | | |
|  | Features | C24-carnitine  C26-carnitine  C24-carnitine /C20-carnitine  C24-carnitine/C22-carnitine  C26-carnitine/C20-carnitine  C26-carnitine/C22-carnitine  C24:0-LPC  C26:0-LPC | | | C24-carnitine  C26-carnitine  C24-carnitine/C20-carnitine  C24-carnitine /C22-carnitine  C26-carnitine/C20-carnitine  C26-carnitine/C22-carnitine  C24:0-LPC  C26:0-LPC | | |
| ZS/XDC | **Model** | **LC-15** | | | **VIP-4** | | |
|  | LC | LC1 | | | LC1 | | |
|  | Features | C20-carnitine  C22-carnitine  C24-carnitine  C26-carnitine  C24-carnitine/C22-carnitine  C24-carnitine /C20-carnitine  C26-carnitine/C22-carnitine  C26-carnitine/C20-carnitine  C20:0-LPC  C24:0-LPC  C26:0-LPC  C26:0-LPC/C22:0-LPC  C26:0-LPC/C20:0-LPC  C24:0-LPC/C20:0-LPC  C24:0-LPC/C22:0-LPC | | | C26-carnitine  C26:0-LPC  C26:0-LPC/C22:0-LPC  C26:0-LPC/C20:0-LPC | | |

*BER- Balanced Error Rate; LC-latent component

Balanced error rates of classification averaged over 5-fold 100x stratified cross validation, area under the curve values for one-vs.-others comparison, and features selection for the 9 sparse PLS-DA models (Control/DDE/X-ALD/ZS models: 4LC-full, 3LC-1-15-1, VIP); (X-ALD vs ZDC models: X-ALD-full, LC-8, VIP-8); ZS vs XDC models: (ZS-full, LC-15, VIP-4)

Abbreviations: Zellweger syndrome (ZS), healthy controls (Control), X-linked adrenoleukodystrophy (X-ALD), non-PD neurological patients (DDE), combined population of ZS, DDE and Controls (ZCD), combined population of X-ALD, DDE and Controls (XCD).
